# The Sequelæ of Enteric Fever—IV

**Published:** 1908-06-27

**Authors:** 


### THE SEQUELÆ OF ENTERIC FEVER—IV.

### AFFECTIONS OF THE NERVOUS SYSTEM.

There is scarcely any other febrile affection, except perhaps epidemic influenza, which may be followed by so many nervous sequelæ as may enteric fever. Some of these are observed during the decline of the fever and during convalescence; others occur some time later. In some cases recovery occurs after many months, whilst others remain incurable.

### Neurasthenia.

It is not surprising that the nervous system does not always recover its normal tone completely. Impairment of nerve power is especially prone to occur in cases where the strain of business or of mental worries of any kind has to be incurred before convalescence has become absolute. Returning to work when the patient is for the first time just able to get to business is much to be deprecated, for there is danger of the recuperating nervous system becoming speedily exhausted again. Neurasthenia in its various forms may supervene. The symptoms may be those of the ordinary well-known types of neurasthenia—cerebro-spinal, spinal, sympathetic, visceral. There may, on the other hand, be special symptoms, for which an organic lesion is not unlikely, although clinically the cases are at present grouped under the heading of neurasthenia. For instance, there may be persistent and severe pains in the back, and sometimes in one or both legs, resembling sciatica or the lightning pains of locomotor ataxia, and described under the name of "*typhoid spine.*" Local pain, allied to local neurasthenia, and designated *topalgia*, is occasionally noticed. Another mischief that may be very troublesome is *acroparœsthesia*—that is, an excessive tenderness on the slightest pressure of the toes, the sole of the foot, or more rarely the dorsal surface of the foot. The reverse of this—namely, more or less persistent anæsthesia of a circumscribed area, is occasionally seen. Local vaso-motor neuroses, with redness, swelling, tenderness, and pain in parts of the upper or lower extremities may also be a trouble.

It seems likely that, though classed together as symptoms of neurasthenia, the pains are due to a definite pathological lesion in the posterior nerve roots and ganglia, whilst vaso-motor symptoms may be associated with definite changes in the cells of Clarke's vesicular column in the lateral horn of the grey matter in the spinal cord.

### Transverse Myelitis; Paraplegia; Hemiplegia.

Distinctive gross lesions of the spinal cord or brain are fortunately rare after typhoid fever. There is no need to speak of acute meningitis here, for not only is it very rare, but also it is a complication of the fever rather than a sequela of it.

There are, however, a certain number of unfortunate cases in which either paraplegia or hemiplegia is a sequel of the fever. In some of these there has doubtless been a predisposing cause for this form of mischief, such as spinal caries or syphilis. Sometimes, however, the enteric fever is itself responsible for the trouble, and in this it is by no means unique. Hemiplegia is well known as a possible sequela of other specific fevers, scarlatina for example.

The pathology of both the transverse myelitis and of the hemiplegia is probably identical in kind though different in locality. In each case the lesion is a thrombosis of arterioles. If the artery which clots is a branch of the middle cerebral, hemiplegia results; if on the other hand the affected vessel is in the spinal cord a transverse disc of spinal softening results, and the symptoms are those of paraplegia. The effects are not dissimilar to those of syphilis; the latter may cause either hemiplegia or paraplegia, in each case the result of vascular thrombosis. Of the two lesions, the prognosis is much better for the hemiplegia than it is for the paraplegia. The anatomical arrangement of the blood-vessels in the brain is more favourable to a collateral circulation than is that in the spinal cord; besides which, owing to the cerebrum being a double organ, the unaffected hemisphere can compensate for a lesion in the other in a way which is scarcely possible in the case of the cord. A post-typhoidal hemiplegia may be complete, and yet entire recovery is possible, and very considerable improvement is to be expected. A post-typhoidal paraplegia, on the other hand, is likely to resolve very imperfectly if it has once been complete.

### Disseminated Myelitis; Disseminated Sclerosis.

Instead of a transverse myelitis, only small portions of the cord may become softened, either in one spot only, or at several different spots throughout the cord. The clinical manifestations of local or disseminated myelitis are not only very various, but they are often very obscure. It has been suggested above that some at least of the symptoms usually attributed to spinal or sympathetic neurasthenia may really be due to a focal lesion in the cord. In a quite small proportion of cases the focal lesion may be such that a more definite clinical picture results.

The inflammation, for instance, may be confined to the anterior horn of grey matter, in which case the picture will be precisely like that of acute anterior poliomyelitis of non-typhoidal origin.

A precisely similar affection of the grey matter in the medulla oblongata has been described by Eisenlohr. In three cases he observed symptoms of bulbar paralysis affecting the lips and tongue, interfering with articulation, and accompanied by weakness in the muscles of the trunk. One of the three cases ended fatally, and numerous typhoid bacilli were found in the brain, medulla, cord, and optic chiasma.

Disseminated sclerosis may be the result of acute disseminated myelitis; but besides this it may arise, apparently spontaneously, without any evidence of there having been an acute stage, and often not until many months or years after the typhoid fever has been recovered from. Nevertheless, there seems to be a causal connection between specific fevers and disseminated sclerosis in some instances; Dreschfeld stated that it is found more often after enteric fever than after any other infectious disease.

